# On the Salutary Effects of Topical Cold, in Two Cases of Strangulated Scrotal Hernia

**Published:** 1809-09

**Authors:** Robert Kinglake

**Affiliations:** Taunton


### *On the salutary Effects of Topical Cold, in Two Cases of Strangulated Scrotal Hernia.* By Dr. KINGLAKE.

ANXIOUS to avail myself of every opportunity afforded by my professional experience of recording important practical facts, I have thought it right to submit to public notice the subsequent Cases of Strangulated Scrotal Hernia, in which, prompt and decided relief was obtained by a sudden application of cold to the scrotum and adjoining parts.

A man in this town, aged about 50, had for several years been subject to an occasional descent of a portion of intestine into the scrotum. It at first, as in similar cases, shewed itself in the groin, in the usual situation of inguinal hernia, from thence it progressively advanced until it reached the scrotum, from whence, the patient had in general been able by his own exertions to return it into the cavity of the abdomen; he had also habitually worn a truss to obviate its renewed descent; but in the instance under consideration, either the truss failed in doing its office, or the exertion of the patient in walking a long journey, prevented it from affording the accustomed support, so that the intestine descended in an unusual quantity, and soon became painfully distended and severely strictured at the abdominal ring. The patient made every effort to return it, but could not accomplish it. The pain and swelling now rapidly increased, accompanied with vomiting and rigors. In this state of the case, Mr. Bagster, an ingenious surgeon of this town, was called to the patient's assistance, who after fruitlessly exerting his best endeavours to reduce the prolapsed part, had determined on injecting a tobacco-smoke clyster. The extreme torture the patient suffered, and the imminent danger that existed from the violence of the attending inflammation, occasioned my being consulted before any thing further was done. On seeing the prodigiously increased size and inflamed state of the scrotum, and learning that the effect of topical cold had not been tried, I directed it to be very freely aplied.

Mr. Bagster immediately envelloped the scrotum in a large coarse cloth dripping from a bucket of cold water standing in the room. The patient violently shuddered on the water coming in contact with the inflamed part, and the volume of the swelling instantaneously diminished under its impression; perceiving this effect, I applied plied one hand to the basis of the scrotum, and moderately pressed the hernial tumour with the other from below upwards, in the direction of the abdominal ring, and in less than one minute the prolapsed intestine returned into the cavity of the abdomen, occasioning by the suddenness with which it passed an audible sound. The patient was immediately freed from all pain, the stomach became settled, and no farther complaint of any sort was made. He has since, (now several months) remained in perfect health, and has prevented any farther descent of intestine by cautiously wearing a suitable truss.

In this case, the prolapsed intestine was so tightly compressed by the abdominal ring, and the quantity that had descended was so large, that the reduction of it, without lessening its bulk, appeared to be absolutely impracticable. The medicine given with a view to move the bowels could not be retained on the stomach, every thing swallowed was almost instantly rejected, which occasioned much additional pain in the incarcerated part; nor was it probable that clystering would have afforded the desired relief. The topical cold answered every purpose that could be wished. Not two minutes intervened from its application, to the complete replacement of the fallen intestine.

The other Case of the advantages of topical cold in strangulated hernia, occurred since that which has been recited. The subject of it was a middle-aged man; it had also existed as an habitual scrotal hernia for some time, but how long I do not know.

The patient had occasionally returned it without difficulty; but in the instance referred to, he could not render himself the accustomed relief. Tension and pain occurred at the abdominal ring, the prolapsed portion of intestine became vastly distened with air, forming with the scrotal and inguinal integuments an enormous swelling. In this state, Mr. George Norman first, and afterwards Mr. William Norman, both respectable surgeons at Langport, were called to the patient's assistance. These gentlemen had, conjointly, with much address, unavailingly tried the accustomed modes of relief during several hours. The case becoming hourly more threatening, by the increasing violence of inflammatory pain and tumefaction, I was desired to visit the patient in consultation. Finding, on inquiry, that topical cold had not been in the number of the means that had been fruitlessly employed, it was, at my recommendation, immediately resorted to. Cloths soaked in a pail of cold water, were repeatedly applied to the tumefied fied part, and also to the thighs and legs, during about half an hour. The tensive hardness of the swelling soon diminished, and within an hour became so reduced, as to admit of the patient himself forcing back the prolapsed part by the lateral pressure which he made on the tumour by approximating his thighs, a mode in which he had been accustomed to effect its return on less difficult occasions. The re-placing of the hernial parts within the cavity of the abdomen, afforded the pain-worn patient immediate ease. The stomach ceased to be affected, and the pulse quickly exchanged its small, hard, and rapid beat, for a full, soft, and slow action.

In both these cases, topical cold proved the very remedy that could be desired. It was physically impossible, in either instance, to have effected the reduction of the protruded parts, without having previously diminished the tensive magnitude of the swelling. The stricture at the abdominal ring had the feel of unyielding solidity; the whole volume of the tumour was indeed most unimpressibly hard. The pain of the hernial swelling was extremely acute, with which the stomach sympathised by frequent efforts to vomit, whilst the heart and arterial system were agitated by rapid and unequal movements. Under these circumstances, stimulating the intestinal canal by whatever means effected, could hardly be supposed capable of liberating the confined parts. If intestinal excitement could avail, it already abounded in a distressing degree, and extended its morbid influence to the upper particle of the alimentary canal. No extent of bleeding compatible with life was likely to have diminished the scrotal swelling sufficiently to have admitted of the desired reduction. The powerful influence of topical cold, therefore, in the cases detailed, holds out a decided recommendation of the practice in similar instances.

That which experience has ascertained with regard to what the topical application of cold water is capable of effecting in cases of extreme hernial tumefaction, a just estimate of the nature of the circumstances forming the difficulty would have suggested as highly probable. The redundant heat prevailing in those painful cases, at once dilates the solid parts, and greatly rarifies the air contained in the cavity of the prolapsed intestine, the united effect of which goes to occasion the irreducible enlargement referred to. Topical cold in those instances acts salutarily by transferring heat, and thus contracting the expanded dimension into which the dilating influence of inflammatory tory tory temperature had forced the hernial parts. The solid parts would in consequence contract, whilst the aerial contents of the prolapsed intestine would be condensed, and thus facility would be afforded to the reduction of parts which could not be returned in their undiminished bulk.

The operation of dividing the abdominal ring for the replacement of hernial parts, should on no occasion be resorted to before having persevered in the free application of cold water for at least one hour, during which time every collateral assistance should be also employed to forward the desired reduction. It deserves to be regarded as a practical rule, in cases where patients have been in the habit of occasionally returning by their own dexterity such hernial prolapsions as may befal them, that in difficult instances, after the tension and bulk of the swelling may have been somewhat diminished by topical cold, but not sufficiently to admit of immediately replacing its contents, that the patient should be directed to endeavour to effect the reduction in what may have been the accustomed manner. The experience derived from former success, added to the sensations of the patient, will be the surest guide to the best mode of making the most advantageous exertions for relief.

Topical cold in cases of irreducible hernia is not proposed as a new remedy. It probably has been often employed; indeed, it is only to be wondered that it is not become the established practice. The want of confidence in its efficacy, must have arisen from the insufficient manner in which it has been used. Its anti-inflammant powers are great, and it may be presumed will in general be found adequate to reducing within sufficient limits, the inflammatory enlargement attending hernial stricture.

Although the preceding instances relate more particularly to long standing hernia, yet it may be justly inferred, that the treatment would be equally effectual in cases of a recent descent of any portion of the contents of the abdomen through the abdominal ring.

The inflammatory irritation produced by the increasing stricture on the prolapsed parts is similar, whether the case be new or old. The only material difference is, that in the one case, the aperture of the abdominal ring has been rendered by repeated distension more capacious, and permits a larger quantity of parts to be protruded before painful compression be induced on them than the other; but when this has been produced, the necessity for relief is the same, and the danger of delay equally imminent. In recent cent cases, therefore, as well as in those of long standing, the remedy deserves to be unweariedly applied; for although it should be eventually found not to have availed in rendering reduction practicable, yet it will at least have been importantly serviceable in repressing the progress of inflammation, in obviating gangrene, and in increasing the prospect of success from dividing the abdominal ring.

I would in these cases advise, after a sufficient trial of topical cold, that the patient should be put into a general cold bath, and be there retained in a suitable position for being aided in pressing back the prolapsed part for ten minutes at a time, and that if reduction should not have been effected, to repeat the cold bathing at intervals of half an hour, during at least three or four hours, before the attempt to relieve in this way should be abandoned as hopeless. The warm bath usually had recourse to on these occasions, by stimulating and distending the prolapsed part, is more likely to increase than to diminish the difficulty of reduction; and would rather tend to expedite than to retard the accession of gangrene. It sometimes indeed relieves pain, but (as in cases of enteritis) this is perhaps effected by destroying the little tone on the affected part that prevented the lifeless or gangrenous state from actually occurring.

If an adequate use of topical cold in hernial stricture of any description should become a prevailing practice, it is probable, that its decided efficacy will greatly lessen the danger that has hitherto accompanied these cases, whether attempted to be replaced by the hand, or consigned for the same purpose to the precarious aid of the Surgeon's knife.

I am, &c.

ROBERT KINGLAKE.

*Taunton, August* 14, 1809.

