# Errata

**Published:** 1809-10

**Authors:** 


					I'age 1235, line IE), fur most read almost.
28, for particle read portion.

				

